# Diseases of the breast

**DOI:** 10.1054/bjoc.2000.1516

**Published:** 2000-12

**Authors:** J S Vaidya, M Keshtgar, M Baum

**Affiliations:** Department of Surgery, University College London, 67–73 Riding House Street, London, W1P 7LD, UK


British Journal of Cancer (2000) 83(12), 1769–1770
© 2000 Cancer Research Campaign
doi: 10.1054/ bjoc.2000.1516, available online at http://www.idealibrary.com on IDEAL®

### Book Reviews

### Diseases of the breast

Edited by: Jay R Harris, Mark E Lippman, Monica Morrow, C Kent Osborne
Philadelphia, New York: Lippincot–Williams & Wilkins: Philadelphia, New York, 2000, 1152pp, 0-7817-1839-2

Searching National Library of Medicine's PubMed for the keyword 'breast' yields 31 988 new articles between 1996 and 1999. The editors of *Diseases of the Breast* have taken up the challenge of incorporating the most important and relevant bits of this new knowledge into their second edition; and they have done well.

Starting with the excellent chapters on anatomy and development – including its biochemical control – this clinician-oriented book goes straight onto management of disease. The importance of management of benign breast disease cannot be overemphasized. It affects the younger woman and the most important task of the clinician is to reassure the worried young woman without missing the rare sinister pathology. It is well worth noting that although a minority of this group has a cancer, missing it contributes to most lawsuits in the field. Being under the media microscope, it takes courage to reassure these women without practising too much defensive medicine. The algorithms in this chapter are useful, but must be read along with the text, which elaborates on the complexity of the management decisions that cannot be laid down in a simple algorithm. Toe amputation always cures an ingrowing toenail but this is usually not considered the best option. The false-negative rate of a properly conducted triple assessment can be less than 1% and this can save many a women an unnecessary biopsy.

We have known the two breast cancer genes BRCA-1 and BRCA-2 for some years, and recent findings indicate that there probably are many more less penetrant genes that are yet to be identified. Public knowledge of these genes has forced the profession to devise ways to tackle the difficult and delicate issue of managing the worried well. Experts in the field have shared their wisdom at great length, covering these new areas in detail. There are separate and well written chapters on chemoprevention and prophylactic mastectomy. In addition, the non-genetic risks of breast cancer – which are probably modifiable – are discussed. The complex discussion on balancing the risks and benefits of mutilating interventions in high-risk individuals is well presented.

The section on natural history of breast cancer has explored the radical conceptual changes in the last century, including the Halstedian concept of orderly spread of breast cancer, Fisher's view of pre-clinical systemic spread, and the combination/spectrum of these two concepts. The recent publication of the Danish radiotherapy trials, which suggested that in certain groups of women post-mastectomy radiotherapy may actually improve survival in patients receiving chemotherapy, contradicts the cumulated evidence from randomized trials, that has been presented in the Oxford overview several times, that nuances of local treatment do not have a great impact on survival. We were hoping that there would be some enlightening discussion on this paradox – but perhaps this is not a place for novel ideas.

The pathogenesis – genetic and endocrine – of breast cancer, and the various types of pathology, are very well covered in separate chapters. The local treatment options of in situ and invasive breast cancer are described very well and so also are special situations like locally advanced breast cancer, pregnancy and cancer, male breast cancer, lymphoedema and occult primary with axillary metastasis. The sensitive topics like treatment of in situ cancer, evaluation of patients after primary therapy, breast reconstruction and psychosocial aspects are discussed in detail.

In this book, metastatic disease is presented in great detail and takes up nearly a third of the book. There is a full discussion about the pros and cons of follow-up after active treatment is completed and several clinical, economic and psychological issues are well addressed. The standard treatments as well as newer methods of treatment of metastasis have been dealt with extensively. The clinical appearance of distant metastasis many years after treatment of primary tumour has remained a mystery despite all advances in the molecular and clinical research. Intense research about growth factors, angiogenesis, apoptosis and the host–metastasis equilibrium is now reaching the bedside. These newer and complex developments are covered in a lucid manner in the book. Detailed overviews of novel treatment modalities using angiogenesis as a therapeutic target, immunotherapy and biologic as are presented in individual chapters.

The section on breast imaging covers the latest advances in image-guided biopsy techniques. These techniques, especially the vacuum-assisted core biopsy (Mammotome) have brought great change in diagnostic work-up of patients with non-palpable breast lesions, avoiding a surgical biopsy in most cases. The excellent chapter on screening includes a section on 'risk-based' screening guidelines, which unfortunately usually have to be based on expert opinion only. A discussion on the risks of screening and therefore what an informed consent for screening should include is sadly lacking. This is particularly relevant today, with very sensitive imaging technology that has started uncovering more and more 'pseudodisease' that may never have manifested in the woman's lifetime. Physical breast examination should have received more coverage, since this may be the only feasible intervention in developing countries and may have the advantage of being able to identify only the potentially fatal cancers.

There are four useful chapters on research methods, techniques of molecular biology and of conducting clinical trials, and assessment of quality of life and cost-effectiveness. In addition, medico-legal issues have a chapter devoted to them.

*Diseases of the Breast* provides extensive coverage of the whole spectrum of issues in breast cancer, with special emphasis on management, while not losing sight of the basic science and patient-related factors. The extensive referencing of every chapter appears to be editorial policy and many of the classic studies should be very useful, as should be the management summaries at the end of relevant chapters. Colour illustrations, especially for some inflammatory conditions, reconstructive procedures and schemata of molecular mechanisms of disease, would be welcome – perhaps in the next edition.

One of us had reviewed the first edition of *Diseases of the Breast* in 1996 and was very impressed – the second edition is as impressive and the content is thoroughly updated. It can therefore be repeated that ‘this book deserves a place on the desk of anyone interested in the subject’.

*Jayant S Vaidya, Mohammed Keshtgar and Michael Baum*
Department of Surgery, University College London,
67–73 Riding House Street, London W1P 7LD, UK

doi: 10.1054/ bjoc.2000.1517, available online at http://www.idealibrary.com on IDEAL®

### Clinical Oncology, 2nd edn

MD Abeloff, JO Armitage, AS Lichtes and JE Niederhuber

Churchill Livingstone: Philadelphia

This is the second edition of this large textbook, which first appeared in 1995. It is one of several books that aim to give a comprehensive account of the science, clinical features and management of cancer.

The growth of electronic libraries and reference systems in the 1990s worried medical publishers, particularly with respect to books like this. Would clinicians continue to use them? It turns out that they do. The reasons are obvious. It’s much easier to look something up quickly in a book than to turn on your computer and struggle through complex sorting procedures to find information that you then have to read on a screen. Books are easy to browse, to dip into, to find something quickly, to read on a train (although at 4.5 kg you wouldn’t want this one to fall out of an overhead luggage rack). A good book, writen to *educate* rather than to review current knowledge, has a different purpose from a collection of reviews. This one is aimed at trainee and practising oncologists, as well as non-specialists. It is a work of instruction and reference with a wide audience. For the editors and publishers this means achieving consistency of style and content. They have succeeded admirably.

To start with the basics. It is 3000 pages long has 223 contributors, nearly all of whom are from North America. It is nicely printed on paper with little see-through and with figures, tables and colour prints that are, in the main, well thought out and with a certain consistency and logic from chapter to chapter. The general layout is conventional. It starts with the scientific underpinnings of cancer medicine, moves on to the general principles of management. It correctly places supportive care early in the book rather than after the sections on specific cancers that follow.

I always find the science parts of these books less useful than the clinical. They are usually mini-reviews and often do not deal conceptually with the subject at a level which will really engage the reader. The authors, basic scientists of high repute, often show little understanding of what the reader will wish to know, or where he or she might have difficulty in understanding. If the function of these sections is to teach and to explain, the text must concern itself with the clear exposition of principles. In this book many of the contributions are first-rate from this point of view. These chapters have good diagrams to explain difficult ideas. It’s curious, though, that there is cumulatively much more space devoted to the science of potential biological treatments – most of which are of uncertain value – than there is to the scientific basis of drug treatment – which often works. As usual there are hundreds of references in these and the other chapters. These have become an obsession in medical writing. It seems that you can’t write that patients are anxious about the diagnosis of cancer without giving three references. Who uses these lists? Why don’t we limit ourselves, in textbooks of medical education and practice, to references to really good reviews or essential original articles?

The sections on the general aspects of cancer management – metastases, effusions, cord compression, paraneoplastic syndromes, nausea, cachexia, fever – are excellent. Here, as elsewhere in the book, there are summary boxes and algorithms which will help the physician, especially the trainee. The advice on diagnosis and management is clear and written concisely. I found myself occasionally at odds with some of the recommendations. Mostly this was when advice was being given about treatment, in situations where there is no chance of cure, without the option of not treating being considered. This remark will doubtless be seen as a typical example of UK nihilism and attributed to our health care system. It isn’t. It’s good practice not to give active treatment sometimes.

How does a reviewer give a fair account of the remaining 2000 pages of the specific tumours? I had this book for 6 months before writing this review – to the immense frustration of the Editor of the *Journal*. The reason was that I used it to look things up when I wasn’t sure of my facts. It’s a good test of whether a book actually works I think. It seldom let me down. I found it easy to find my way around. I read the sections on lung cancer, lymphoma, bone and soft-tissue sarcoma and childhood cancer thoroughly, and individual problems in many of the other sections. I found the description of the clinical features to be very good, the advice about diagnosis and staging to be sensible and straightforward, and the approach to management to be up-to-date and clear. There is a tendency for chemotherapy to be given a greater weight in some cancers than the results actually justify, but this probably reflects a feature of North American practice. There was one area of real confusion and that is in the discussion of round cell sarcomas of bone. This has found its way into two chapters, neither of which deals with the management well.

The graphic illustration is clear, the clinical algorithms are useful. The tables of chemotherapy regimens are highly selected (they always are) and reflect the authors’ own preoccupations, but those that I looked at in detail seemed to me to be a fair summary of the most useful modern regimens.

In summary, this is a first-rate book. I congratulate the Editors. I know what a task it must have been to ensure consistency of style and content and to achieve a balance between the chapters. It will be in every oncologist’s library. In my view it is one of the best big books on cancer.

*Robert Souhami*
Royal Free and University College Medical School,
University College London,
Gower Street,
London WCIE 6BT, UK

 *© 2000 Cancer Research Campaign*

